# Intracorporeal anastomosis in right hemicolectomy for colon cancer: short-term outcomes with the DaVinci Xi robot

**DOI:** 10.1007/s11701-020-01188-y

**Published:** 2021-01-25

**Authors:** Søren Rattenborg, Lars Bundgaard, Jesper Andersen, Jan Lindebjerg, Jacob Kuhn, Conny J. Jakobsen, Hans B. Rahr

**Affiliations:** 1 Department of Surgery, Vejle Hospital, University Hospital of Southern Denmark, Vejle, Denmark; 2 OPEN, Open Patient Data Exploratory Network, Odense, Denmark; 3 Colorectal Cancer Center South, Vejle Hospital, University Hospital of Southern Denmark, Vejle, Denmark; 4 Department of Pathology, Vejle Hospital, University Hospital of Southern Denmark, Vejle, Denmark; 5 Institute of Regional Health Research, University of Southern Denmark, Odense, Denmark; 6 Department of Anesthesiology, Vejle Hospital, University Hospital of Southern Denmark, Vejle, Denmark

**Keywords:** Colon cancer, Robotic surgery, Right colectomy, Ileocolic anastomosis

## Abstract

Intracorporeal anastomosis (IA) may improve outcomes compared with extracorporeal anastomosis (EA) in minimally invasive right colectomy. This is a prospective series of robotic right hemicolectomies (RRC) with IA from one institution. 35 consecutive patients with verified or suspected right colon cancer undergoing RRC with IA, and historic control groups of 22 RRC and 40 laparoscopic right colectomies (LRC), both with EA. Primary outcome measure was length of stay (LOS). Secondary outcome measures were 30-day complication rates, readmissions, pain scores, analgesic consumption, and specimen quality. Median LOS did not differ significantly between the groups (RRC-IA, 4 days; LRC-EA, 4 days; RRC-EA, 5 days). In-hospital surgical complications Clavien–Dindo 3 + were seen in 1, 2, and 0 patients, respectively, and 3, 5, and 3 patients were readmitted to hospital within 30 days. Median pain score was 2 in all groups on postoperative day (POD) 2. Relatively more patients in the RRC-IA group received gabapentin on POD 2 (*p* = 0.006), but use of other analgetics did not differ between groups. Mean specimen lengths were 31, 25 and 27 cm, respectively (RRC-IA vs. LRC-EA, *p* = 0.003), but mesentery width, proportion of mesocolic excisions and number of lymph nodes did not differ between the groups. RRC-IA was not associated with shorter LOS, fewer complications or better specimen quality than recent controls undergoing either RRC-EA or LRC-EA.

## Introduction

In recent years laparoscopic right hemicolectomy (LRC) with extracorporeal anastomosis (LRC-EA) has become the standard treatment for right-sided colon cancer. The change in operative technique has been accompanied by other improvements, e.g. enhanced recovery programs (ERAS) [1], and length of stay (LOS) as well as short-term morbidity and mortality have improved [2]. Nevertheless, colorectal surgery still entails substantial short term morbidity [3].

It has been suggested that constructing the anastomosis laparoscopically within the abdomen (i.e. intracorporeal anastomosis, IA) could improve outcomes even further by avoiding exteriorization of the bowel. The laparotomy, which is known to be a main trigger of surgical stress, may be minimized to a size just enough to allow for extraction of the specimen, and placed just above the pubis, i.e. Pfannenstiel incision. This may potentially reduce surgical stress [4], pain and adverse effects on respiration, and thus, ultimately, LOS.

It may also be speculated that a longer specimen with less defects and tears can be obtained, and less stretching and damage to the mesentery and its vessels might also lead to fewer complications, especially in obese patients.

Performing IA safely with conventional laparoscopic technique is, however, rather difficult and time consuming [5, 6]. This may potentially be overcome by robot-assisted right hemicolectomy (RRC).

Recent comparisons of laparoscopic IA and EA have shown IA to be safe with less short-term morbidity and decreased LOS [7, 8] but Allaix et al. [5] showed a higher, albeit non-significant, rate of anastomotic leakage. Most studies do not include ERAS principles. This study aimed to determine LOS and other short-term outcomes as well as specimen quality in a cohort of consecutive patients undergoing robotic assisted right hemicolectomy (RRC-IA) for colon cancer and comparing these with previous RRC-EA and LRC-EA procedures from the same institution.

## Materials and methods

### Design

A single-arm, single-center prospective study of consecutive patients with historical controls.

### Patients and ethics

Legally competent patients with verified or suspected right colon cancer, planned for elective robot-assisted right hemicolectomy with curative intent, could be included in the study group after oral and written informed consent. A sample size calculation based on 97 previous patients suggested that 35 patients would be needed to show a reduction of length of stay (LOS) by 2 days. In addition, five patients were recruited for an initial pilot series for testing and aligning the study setup. These 5 patients were required to have a body mass index (BMI) ≤ 30 and were not part of the study group. The historical controls were recent cases of RRC-EA and LRC-EA, respectively.

### Before surgery

On the day before surgery all patients received Bisacodyl 10 mg in the morning and afternoon and only fluids by mouth, along with an enema on the day of surgery as bowel preparation. Regular consumption of analgesics prior to surgery was registered. All patients were routinely allocated by a local risk stratification system (RITA) to one of three levels of standardized intra- and postoperative care according to department guidelines and ERAS principles.

### Surgery

**Fig. 1:**
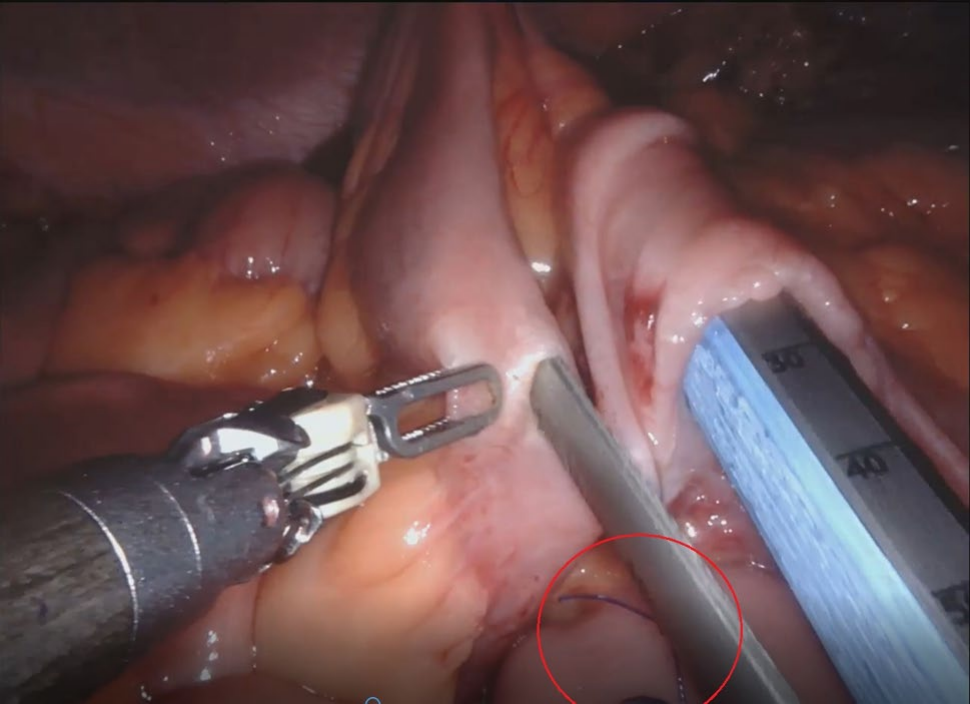
An intracorporeal anastomosis being created. Stay suture (red circle). Da Vinci™ Endowrist® stapler is inserted through enterotomies in ileum (left side) and transverse colon (right side)

RRC-IA was performed by either of two fellowship-trained high-volume laparoscopic colorectal surgeons. Both had experience with 70 + robotic colectomies on the DaVinci Xi robot and 170 + colorectal resections prior to this study. Although experienced with EA, they had little experience (< 5) with IA, but had studied the technique in Denmark and abroad. The 35 RRC-IA cases were done by one of the two study surgeons with an experienced assistant. The operation was performed in a standardized fashion. After mobilization of the right colon, the transverse colon and ileum were divided by DaVinci blue-cartridge robotic staplers 45 mm. After isoperistaltic alignment of the colon and ileum side-to-side, stay sutures were placed. The same robotic stapler with a blue cartridge was introduced through enterotomies, and a linear anastomosis as long as the stapler was created. Enterotomies were closed in two layers with 3–0 V-Loc™ suture (Fig. 1).

The site and length of the laparotomy for specimen extraction were recorded; at the fascial level after removal of the specimen, and at skin level after closure of the skin.

Operating time was defined as time in minutes from first incision until the skin was closed.

### Postoperative care

All postoperative care and observations were according to department routine. This allows for meaningful comparisons between the three groups.

Postoperative pain was monitored using visual analog scale (VAS) score, from 0 to 10, in the morning and recorded in the patient’s electronic medical record (EMR) along with consumption of pain medication. Medical and surgical complications and readmissions within 30 days after surgery were registered. Any readmissions outside the Region of Southern Denmark were not available for registration.

Patients were discharged from hospital when they fulfilled the following discharge criteria: Full enteral nutrition, flatus and bowel motions, walking around or at least mobilized like before surgery, on oral analgesics only, with no signs of wound infection, and voiding freely.

### Data analysis

Prospective data were extracted from the patients’ EMRs and entered into a custom-built database on the REDCap platform. These data were enriched by additional clinical and pathoanatomical data from the Danish Colorectal Cancer Group (DCCG) database. Any missing or unexpected data were checked against the EMR or pathology reports. All complications and readmissions were reviewed and validated by the senior authors (LB, JA, HBR), and any disagreements were resolved through consensus.

The primary outcome measure was length of stay (LOS), defined as the number of days from the day of surgery (postoperative day (POD) 0) until the day of discharge from hospital. In cases where discharge from hospital was postponed for ethical or practical reasons, both dates—the day on which the patient was deemed ready for discharge, and the day of actual discharge—were recorded.

Secondary outcome measures were complications as recorded in the DCCG database and graded according to Clavien–Dindo [9], readmissions within 30 days after surgery, pain scores, analgesic consumption and specimen quality. Pain scores and analgesic consumption were analysed on POD 2, when any residual effect of intraoperative medication was considered negligible and most patients were still in hospital. Consumption of analgesics was calculated as the dose on POD 2 after subtraction of any preoperative regular medication of similar type, i.e. as the amount of extra analgesics needed by the patient. Opioid doses were converted to morphine equivalents for comparison. Resected specimens were handled according to national guidelines, i.e. they were sent immediately to the Pathology Laboratory, where they were measured and photographed before fixation. Specimen quality was judged by overall specimen length, perpendicular distance from the vascular tie to the bowel wall, plane of resection, and number of lymph nodes.

Descriptive statistics were used. The three patient groups were compared by one-way ANOVA and pairwise comparisons with Tukey’s correction for normally distributed data, and by Kruskal–Wallis and Wilcoxon tests for non-normally distributed data. STATA 15 was used for all statistics. *p* < 0.05 was considered statistically significant.

## Results

From 15th Nov 2017 to 5th Dec 2018, 43 patients were considered for inclusion. One patient refused to participate, and in three cases, none of the study surgeons were available. Thus, 39 patients were included, of whom 4 were subsequently excluded due to conversion of the operation to open surgery (3 due to unclear anatomy, 1 due to adherent tumor). The remaining 35 patients constitute the study group (RRC-IA). For the control groups, 22 RRC-EA and 40 LRC-EA were available from the years 2015–2018.

Baseline characteristics of the three groups are shown in Table 1.

**Table 1:** Baseline characteristics of 97 patients undergoing minimally invasive right hemicolectomy with intracorporeal or extracorporeal anastomosis

		RRC-IA^1^ (*n* = 35)	LRC-EA^1^ (*n* = 40)	RRC-EA^1^ (*n* = 22)
Gender	Female (%)	19 (54%)	25 (63%)	13 (59%)
Age (years)	Median (range)	74 (53–92)	73 (47–89)	71 (49–93)
Body mass index (kg/m^2^)	Median (range)	27.4 (18.6–43.1)	25.5 (20.4–37.1)	25.5 (17.4–31.1)
Pathology (no. of patients (%))				
Primary colon cancer	UICC stage I	6 (17%)	12 (30%)	3 (14%)
	II	8 (23%)	14 (35%)	6 (27%)
	III	10 (29%)	9 (23%)	12 (55%)
	IV	2 (6%)	4 (10%)	1 (5%)
Other cancer^2^		4 (11%)	–	–
Benign		5 (14%)	1 (3%)	–
Charlson score from DCCG^3^ (no. of patients (% of non-missing))	0	12 (48%)	21 (53%)	11 (50%)
	1	5 (20%)	3 (8%)	5 (23%)
	2	5 (20%)	5 (13%)	4 (18%)
	3+	3 (12%)	11 (28%)	2 (9%)
	Not in DCCG	10	–	–
Performance score from DCCG (no. of patients (% of non-missing))	0	17 (68%)	33 (83%)	14 (64%)
	1	7 (28%)	4 (10%)	7 (32%)
	2	1 (4%)	–	–
	3+	–	2 (5%)	1 (5%)
	Unknown	–	1 (3%)	–
	Not in DCCG	10	–	–

^1^*RRC-IA* robotic operation with intracorporeal anastomosis, *LRC-EA* laparoscopic operation with extracorporeal anastomosis, *RRC-EA* robotic operations with extracorporeal anastomosis

^2^Appendiceal cancer (2), metachronous cancer (1), neuroendocrine tumor (1)

^3^The Danish Colorectal Cancer Group national database

### Length of stay, in-hospital complications

**Fig. 2:**
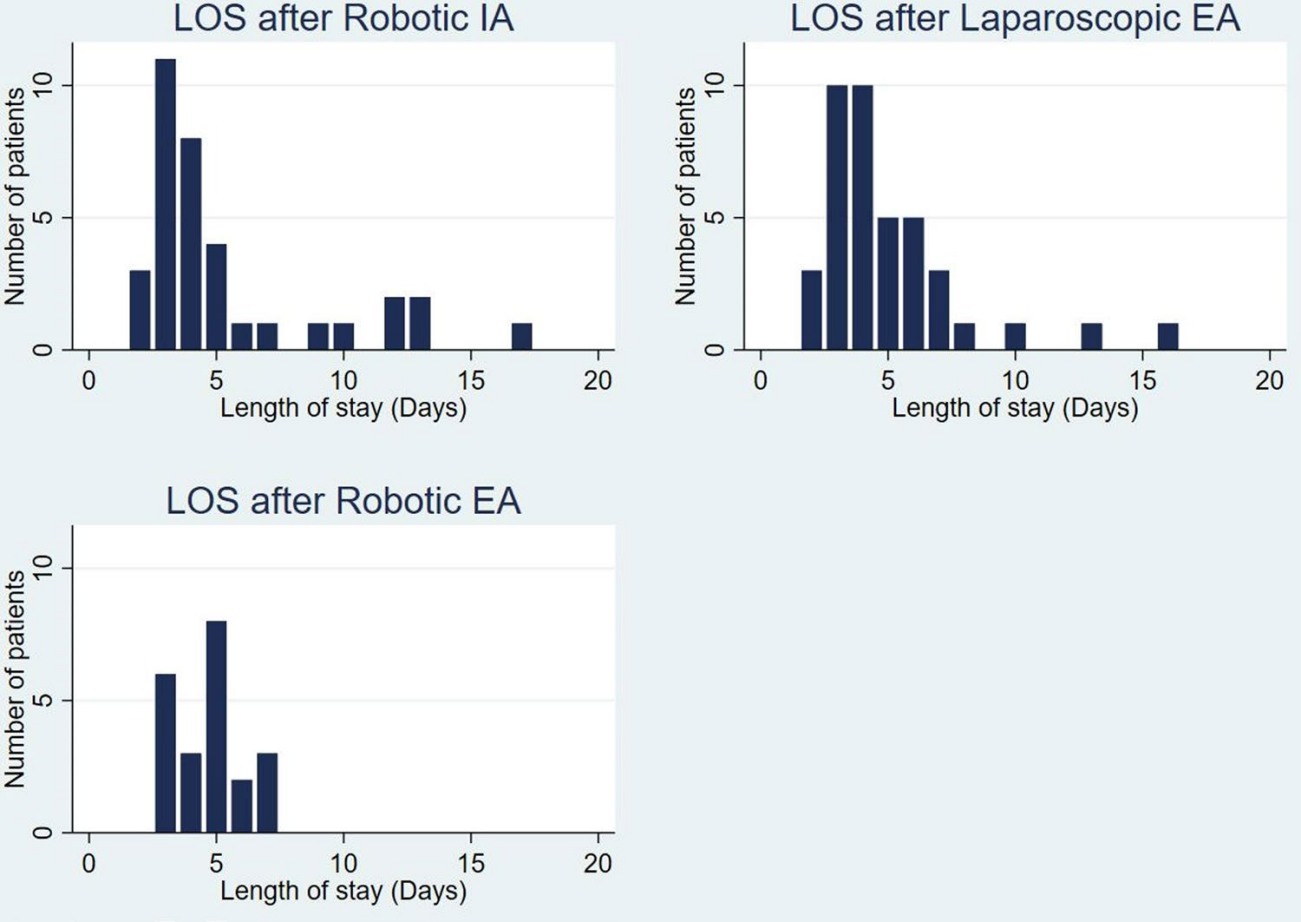
Distribution of length of stay (LOS) of 97 patients undergoing minimally invasive right hemicolectomy with intracorporeal or extracorporeal anastomosis. *IA* intracorporeal anastomosis, *EA* extracorporeal anastomosis

As shown in Table 2, median LOS did not differ significantly between the groups. In the RRC-IA and LRC-EA groups, distribution of LOS was strikingly right-skewed as compared with the RRC-EA group (see Fig. 2). Five of the seven RRC-IA patients with LOS > 7 days had bowel paralysis > 4 days, which resolved spontaneously, as shown in Table 2. Excluding these five from analysis did not change the median LOS. Bowel paralysis was not observed in the RRC-EA group. Five patients were discharged later than the day they were deemed ready for discharge (not shown). Using the latter dates did not change the medians. None of the medical, and only few of the surgical, complications were graded Clavien–Dindo 3 + (see Table 2). Anastomotic leakage was recorded in one patient in the RRC-IA group. At the reoperation, a defect was found in the stapler line closing the colonic end, but the anastomosis itself was intact, and a new side-to-side anastomosis was created.

**Table 2:** Outcomes in 97 patients undergoing minimally invasive right hemicolectomy with intracorporeal or extracorporeal anastomosis

		RRC-IA^1^ (*n* = 35)	LRC-EA^1^ (*n* = 40)	RRC-EA^1^ (*n* = 22)
Length of stay until discharge (days)	Median (min–max)	4 (2–17)	4 (2–16)	5 (3–7)
*In-hospital complications, medical*				
Pneumonia	No. of patients	1	1	1
Heart failure		–	1	–
Arrhytmia		1	–	1
Arrhytmia + urinary tract infection		1	–	–
Syncope		–	–	1
Clavien–Dindo ≥ 3		–	–	–
*In-hospital complications, surgical*^2^				
Bleeding	No. of patients	1	–	–
Bowel paralysis/ileus		5	1	–
Wound abscess		–	1	–
Anastomotic leakage		1	1	–
Other		4	2	1
Clavien–Dindo ≥ 3		1	2	–
*Readmissions ≤ 30 days after surgery*				
Medical complication	No. of patients	2	2	–
Clavien–Dindo ≥ 3		–	–	–
Surgical complication		1	3	3
Clavien–Dindo ≥ 3		–	1	–

^1^*RRC-IA* robotic operation with intracorporeal anastomosis, *LRC-EA* laparoscopic operation with extracorporeal anastomosis, *RRC-EA* robotic operations with extracorporeal anastomosis

^2^Bowel paralysis was defined as nasogastric tube or absence of bowel motions after POD 4. Other in-hospital complications were: urinary retention (3 RRC-IA), rectal bleeding (1 RRC-IA), radiological pneumoperitoneum, but laparotomy with no pathology (1 LRC-EA), serous discharge from port site managed laparoscopically (1 LRC-EA) or conservatively (1 RRC-EA)

Three patients in the RRC-IA group were discharged with an indwelling bladder catheter.

### Readmissions

In the RRC-IA group, two patients (6%) experienced one or two readmissions for a medical complication (pneumonia, arrhythmia, dehydration), and one patient (3%) a readmission for rectal bleeding under anticoagulant therapy. None were graded Clavien–Dindo > 2.

### Pain and analgetics

VAS score on POD 2 was missing in 11 patients (RRC-IA, 5; RRC-EA, 4; LRC-EA, 2). Two patients had an epidural catheter on POD 2. Along with six patients who were discharged on POD 2, these two were excluded from analysis of analgesic consumption.

On POD 2 median VAS score in the morning was 2 for all groups.

On POD 2, 76/85 (89%) patients received additional paracetamol [RRC-IA, 90%; LRC-IA, 89%; RRC-EA, 90%; not significant (n.s.)]. Two patients in the LRC-EA group received additional NSAID. Additional gabapentin was given to 59/89 (66%) (RRC-IA, 88%; LRC-EA, 56%; RRC-EA, 52%; *p* = 0.006). Additional opioids (morphine, oxycodone, tramadol, codeine) were administered to 33/86 (38%) on POD 2 (RRC-IA, 29%; LRC-EA, 43%; RRC-EA, 45%; n.s.).

### Specimen quality

On average, specimens were 4–6 cm longer in the RRC-IA group than in the two other groups, but in a pairwise comparison only the difference between RRC-IA and LRC-EA reached statistical significance. Mesentery width, plane of resection, and number of lymph nodes did not differ between the groups (Table 3).

**Table 3:** Specimen quality in 97 patients undergoing minimally invasive right hemicolectomy with intracorporeal or extracorporeal anastomosis

		RRC-IA^1^ (*n* = 35)	LRC-EA^1^ (*n* = 40)	RRC-EA^1^ (*n* = 22)
Specimen length (unfixed)	No of specimens	27	35	20
	Mean length, cm (95% CI)	31 (28–34)	25 (23–27)	27 (24–30)
Mesentery width (unfixed)	No of specimens	22	35	20
	Median width, mm (range)	94 (55–160)	90 (30–152)	89 (28–135)
Plane of resection	No of specimens (% of non-missing)	34	40	22
Mesocolic		27 (79%)	32 (80%)	19 (86%)
Intramesocolic		7 (21%)	7 (18%)	3 (14%)
Intramuscular		–	1 (3%)	–
Lymph nodes	No of specimens	29	40	22
	Mean no. of lymph nodes (95% CI)	36 (32–40)	36 (32–40)	36 (30–41)

^1^*RRC-IA* robotic operation with intracorporeal anastomosis, *LRC-EA* laparoscopic operation with extracorporeal anastomosis, *RRC-EA* robotic operation with extracorporeal anastomosis

### Other observations

Operating time was significantly longer with both robotic methods (RRC-IA, mean 153, 95% CI 142–165 min; RRC-EA, mean 138, 95% CI 125–151 min) than with LRC-EA (mean 104 min, 95% CI 94–113 min). The mean difference between RRC-IA and RRC-EA was 15.6 min (n.s.).

Intraoperative blood loss was 50 ml in the RRC-IA group, and did not differ between the three groups.

Mean length of the Pfannenstiel incision in the RRC-IA group was 7.8 cm at skin level and 7.4 cm at fascial level.

## Discussion

This study aimed to elucidate the potential advantages of RRC-IA in terms of short-term outcomes and specimen quality during the implementation of the technique in a medium-sized colorectal unit to assess whether the increased cost of using the robotic stapler may be justified.

We could not demonstrate any improvement regarding the primary outcome measure, length of stay. On the contrary, some of the patients in the RRC-IA group had prolonged hospital stays due to bowel paralysis, a complication not seen in the RRC-EA group. Previous non-randomized comparisons of IA and EA in laparoscopic [7, 10] and robotic [11, 12] surgery have reported shorter LOS after IA, but with mean differences of only 0.5–1.1 days, and with statistical significance almost exclusively in larger series or meta-analyses. The only randomized trial known to us compared laparoscopic (not robotic) IA and EA and found no difference in LOS [5]. We have no explanation for the bowel paralysis seen in five of the RRC-IA patients.

Regarding the secondary outcome measures, i.e. complication rates, readmission rates, pain scores and analgesic consumption, these did not differ markedly between groups. As expected, significantly longer specimens were obtained in the RRC-IA group, but the mesentery width, the proportion of intact mesocolic dissection plane, and number of lymph nodes, did not differ between groups. Operating time was significantly longer in the two robotic groups than in the laparoscopic group.

Strengths of this study were its prospective, consecutive design and the single-center setting with two dedicated surgeons performing all RRC-IA operations. Also, the control groups were rather recent (later than 2015), allowing for a meaningful comparison.

Perhaps the most important contribution of this study is its real-life comparison of RRC-IA with two other commonly used techniques, presenting data on a wide range of outcome measures including relevant aspects of specimen quality. This should provide others considering IA with a fair depiction of what to expect if they choose to implement RRC-IA.

A limitation was the retrospective assessment of the control groups, although most data stem from the DCCG database, which is based on prospective registration. Some treatment principles had also changed with time, as reflected by the differences in analgesic regimes between the groups. In addition, the RCC-IA group might inadvertently have received more attention and care during the perioperative course than the two retrospective control groups. Furthermore, the generalizability of the study is somewhat limited by the setting. Having two experienced robotic surgeons implementing a new technique probably reflects the situation most departments face when deciding to change their practice, but it should be realized that the present series represents the beginning of a learning curve. Outcomes might be different at a later stage when more experience has been gained, or if newcomers to robotic surgery chose to take up RRC-IA [13]. Conversions were excluded to allow comparisons with the control groups (only laparoscopically completed cases were available), but it might be argued that an intention-to-treat design would be better because it allows for comparisons of rates and outcomes of conversions. A final limitation, of course, is the limited number of patients, particularly in the RRC-EA group, and consequent lack of statistical power. Other studies of the same magnitude have also failed to demonstrate any differences in LOS [12].

In our own practice, we have chosen to proceed with RRC-IA because the results suggest that the technique is safe in our hands and holds promise for a better specimen quality. This seems in line with previous studies [8, 12]. The bowel paralysis problem should be addressed in future studies. We expect our short-term outcomes to improve with experience and look forward to report our first 100 cases in the future Fig. 1.

## Conclusion

Robot-assisted right colectomy (RRC) with intracorporeal anastomosis (IA) was not associated with shorter length of stay, fewer complications or better specimen quality than historical controls undergoing either RRC with extracorporeal anastomosis (EA) or traditional laparoscopic right colectomy with EA.

## Data Availability

The datasets generated and analysed during the current study are not available to the public in accordance with the Danish data protection Act. The corresponding author will try to meet any reasonable request, provided that prior permission can be obtained from the Danish Data Protection Agency.
